# Formulation of Polymeric Micelles to Increase the Solubility and Photostability of Caffeic Acid

**DOI:** 10.3390/molecules29143329

**Published:** 2024-07-15

**Authors:** Elisabetta Mazzotta, Martina Chieffallo, Rita Muzzalupo, Miriana Spingola, Paolino Caputo, Martina Romeo, Giuseppina Ioele

**Affiliations:** 1 Department of Pharmacy, Health and Nutritional Sciences, University of Calabria, 87036 Rende, Italy; mazzotta-elisabetta@libero.it (E.M.); martina.chieffallo@unical.it (M.C.); miry_sping97@hotmail.it (M.S.); martina.romeo@unical.it (M.R.); giuseppina.ioele@unical.it (G.I.); 2 Department of Chemistry and Chemical Technologies, Cubo 14/D, University of Calabria, Via P. Bucci, 87036 Arcavacata di Rende, Italy; paolino.caputo@unical.it

**Keywords:** caffeic acid, P123, polymeric micelles, antioxidant activity, photodegradation studies, release studies, skin permeation

## Abstract

Caffeic acid (CA), a hydrophobic polyphenol with various pharmacological activities, exhibits a low aqueous solubility and sensitivity to light. In order to improve its chemical properties and overcome the limits in its application, the compound was loaded in P123 micelles (MCs) prepared using two polymer concentrations (10 and 20% *w*/*w*, MC10 and MC20). The micelles were characterised in terms of the size distribution, zeta potential, drug encapsulation efficiency, rheology, and cumulative drug release. Micellar formulations exhibited sizes in the range of 11.70 and 17.70 nm and a good polydispersion, indicating the formation of relatively small-sized micelles, which is favourable for drug delivery applications. Additionally, the stability and antioxidant profiles of the free CA and the CA loaded in micelles were studied. The results obtained on the free CA showed the formation of photodegradation products endowed with higher DPPH scavenging activity with respect to the pure compound. Instead, it was found that the incorporation of CA into the micelles significantly increased its solubility and decreased the photodegradation rate. Overall, the results indicate the successful formation of P123 micelles loaded with CA, with promising characteristics such as a small size, good encapsulation efficiency, sustained release profile, and improved light stability. These findings suggest the potentiality of these micelles as a delivery system for CA, thus enhancing its bioavailability.

## 1. Introduction

Caffeic acid (CA) (3,4-dihydroxycinnamic acid) is a naturally occurring polyphenol with a low molecular weight isolated from various plants such as coffee, tea, thyme, oregano, sage, berry, apple, and potato [1]. In addition to the widely reported antioxidant activity [2], CA also exhibits various pharmacological properties such as anticancer, antibacterial, hepatoprotective, anti-inflammatory, and antiglycaemic properties [3,4,5,6]. Recently, the therapeutic potential of CA in preventing and treating Alzheimer’s disease by reducing the production and aggregation as well as promoting the clearance of amyloid β peptide has been reported [7]. Despite these potential properties, its application is limited by the high hydrophobicity, poor bioavailability, and high instability to environmental stress [8,9]. It is known that CA has poor photostability and is readily converted into a cis stereoisomer degradation product upon exposure to light [10]. An aqueous CA solution also resulted in being sensitive to oxygen, light, and high temperature, leading to a complete degradation after 30 days of storage [11]. In this context, its encapsulation in nanoscale delivery systems could represent a useful strategy to improve the solubility and control the chemical stability. Among the various nano-delivery systems, polymeric micelles are interesting devices for the delivery of hydrophobic compounds. Polymeric micelles have shell–core structures formed by the spontaneous self-assembly of amphiphilic polymers in water. Other than improving solubility, the encapsulation of hydrophobic drugs in polymeric micelles have been studied for the ability to protect photosensitive drugs from light. Various research groups have indeed reported the reduced photodegradation of chemicals when incorporated in polymeric micelles [12,13,14]. Moreover, polymeric micelles are widely used for their ability to enhance the skin permeation of a wide range of poorly water-soluble molecules [15,16], enhancing drug solubilisation, increasing partitioning into the deeper layers of the skin, and forming a depot into the skin [17]. Among the polymeric micelles, pluronic copolymers have been widely used in the fields of nanomedicine. Pluronics (PEO-PPO-PEO or PPO-PEO-PPO) are the first family of synthetic non-ionic surfactants, designed and manufactured by BASF, recognised as safe and biocompatible materials in the pharmaceutical field and frequently used for their capacity to solubilise lipophilic drugs [18]. In fact, thanks to their amphiphilic nature, they form micelles above the critical micellar concentration (CMC) [19]. Pluronic micelles are employed as drug delivery systems because of their good biocompatibility, biodegradability, and ability to increase solubility, as well as their capacity to improve the circulation time and release drugs at the target sites [20]. P123 is a hydrophobic pluronic used for drug delivery in the brain and in cancer therapy [21,22]. The P123 polymer results are very interesting, since it exhibits a temperature-dependent aggregation behaviour. At low temperatures, it is well-soluble in water regardless of the polymer chains, while, with the increase in temperature, the solubility of P123 decreases and the chains start to aggregate and form micelles with spherical, worm-like, and lamellar structures [23,24]. The aim of the present study was to develop P123 micelles to enhance the aqueous solubility, topical bioavailability, and stability of CA. Micelles were prepared using various polymer concentrations and loading several drug amounts. CA encapsulation was carried out using the direct dissolution method and was confirmed by the physicochemical characterisation of micelle formulations and in vitro drug release studies. Specifically, release experiments and rheological characterisation were performed at two different temperatures considering the variety of different temperature-dependent P123 micelle morphologies. As a last step in the physicochemical characterisation of the proposed formulations, forced degradation experiments were carried out on CA free and loaded in micelles to assess the stability to light and temperature. All of the experiments were conducted according to the International Conference on Harmonization (ICH) rules [25]. Spectrophotometric measurements coupled with multivariate methods were tuned to assess the possible formation of degradation products [26]. Moreover, the antioxidant activity for both the CA free and loaded in micelles was monitored by DPPH tests both on freshly prepared formulations and during the degradation experiments.

## 2. Results and Discussions

### 2.1. Micelle Characterisation

**Figure 1:**
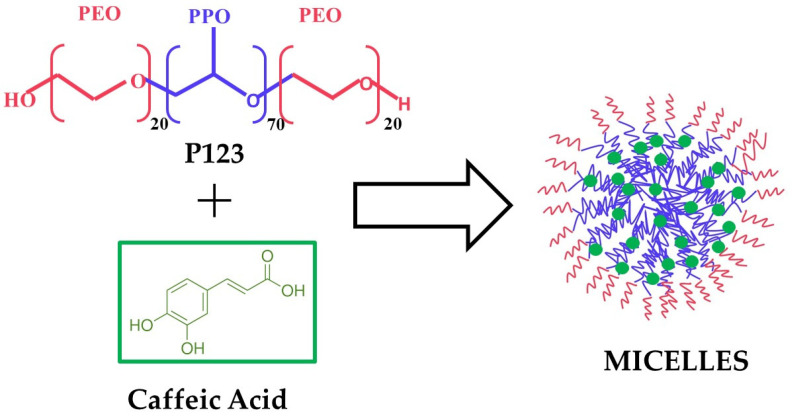
Scheme of the self-assembly of block copolymer P123 micelles loaded with caffeic acid.

In this manuscript, attempts were made to solubilise CA in P123 micelles to increase the bioavailability, stability, and antioxidant activity. Three different micellar aggregates, MC5, MC10, and MC20, were prepared using 5%, 10%, and 20% (*w*/*w*) solutions of P123, respectively. P123 was able to self-assemble in micellar structures and to solubilise hydrophobic caffeic acid according the scheme reported in Figure 1.

The maximum solubilised concentration of CA in the 10% and 20% (*w*/*w*) P123 micelle solutions was 0.5% (*w*/*w*). Conversely, micelles made up of the 5% surfactant were only able to solubilise 0.2% (*w*/*w*) of the CA. For this reason, the micellar solution prepared with 5% of the surfactant (MC5) was eliminated from the subsequent study. The average size for the empty micelles and drug-loaded micelles are reported in Table 1. The mean diameter of the empty P123 micelles and drug-incorporated micelles was in the range between 12.16 and 16.09 nm, with a rather narrow size distribution, as shown in Figure S1, Supplementary Materials (a PI lower than 0.218). Loading the micelles with CA did not visibly affect their size, and the EE% was found to range from 77.68 to 82.59%. Considering the low water solubility of CA, the incorporation into micellar dispersions made it possible to formulate aqueous solutions with increased drug solubility, which can be advantageous for various pharmaceutical applications where an improved solubility can enhance bioavailability.

### 2.2. In Vitro Diffusion and Permeation Studies

One of the primary objectives of this study is to enhance the solubility and improve the drug release capacity of lipophilic molecules. Therefore, the drug release performance of P123 micelles was evaluated by performing diffusion and permeation studies using Franz diffusion cells.

The studies were carried out at two different temperatures, 32 and 40 °C, to evaluate the relation of cumulative CA release from these micelles with respect the increase in temperature. Considering the varying aggregation behaviour of P123 with the temperature, temperature could significantly affect the release properties of the P123 aggregates.

**Figure 2:**
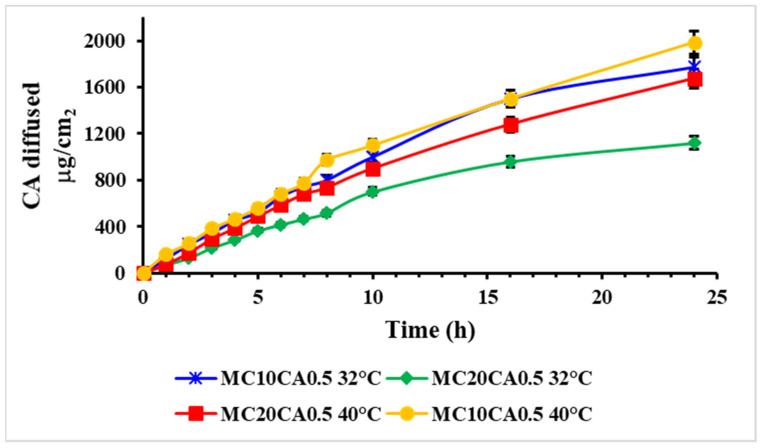
In vitro skin diffusion profiles of CA from MC10CA0.5 and MC20CA0.5 at 32 °C and 40 °C. Results are presented as mean ± SD.

The in vitro diffusion profile of the CA micelles was investigated using Franz diffusion cells at 32 °C and at 40 °C for 24 h. As shown in Figure 2, it was concluded that the diffusion clearly decreased with the increase in the polymer concentration. 

When increasing the P123 amount to 20%, the micelle amount also increased, but the CA percentage was the same in both formulations, and this aspect led to different concentration gradients and, hence, different diffusion coefficients. In fact, the micelles made up of 10% of P123 exhibited a higher amount of diffused drug compared to the MC20CA0.5 ones. The temperature increase, instead, led to a faster CA diffusion for both formulations, and the MC20CA0.5 was more influenced by the change.

**Figure 3:**
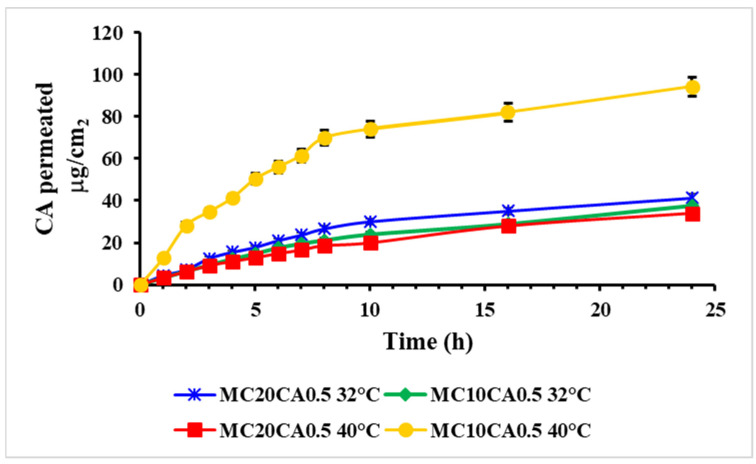
In vitro skin permeation profiles of CA from MC10CA0.5 and MC20CA0.5 at 32 °C and 40 °C. Results are presented as mean ± SD.

**Figure 4:**
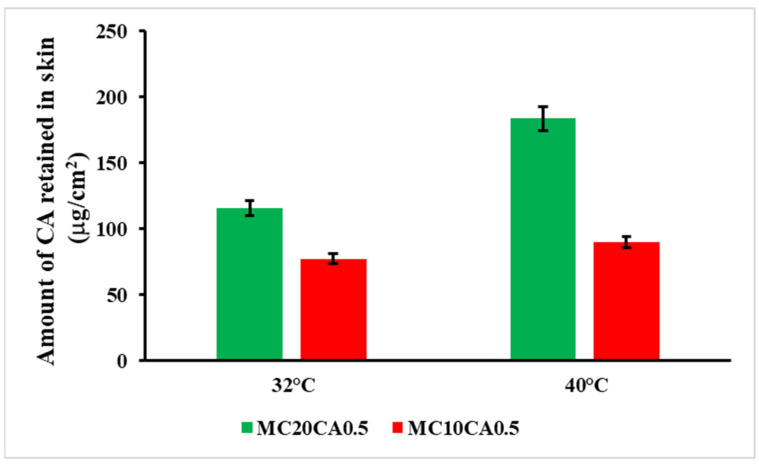
CA retention in rabbit ear skin after exposure to MC10CA0.5 and MC20CA0.5 for 24 h. Results are presented as mean ± SD.

A Franz skin permeation study was also performed to evaluate the permeability of the CA from various formulations at 32 °C and 40 °C. At 32 °C, the permeation profile of the CA from MC10CA0.5 and MC20CA0.5 was similar, while it was significantly higher for MC10CA0.5 at 40 °C (Figure 3). Moreover, the temperature increase led to a higher permeation for the MC10CA0.5 sample: in fact, the amount of CA permeated at 40 °C was equal to 94.18 μg cm^−2^, which resulted to be two-and-a-half times higher with respect the amount permeated at 32 °C, which was equal to 37.76 μg cm^−2^. This trend was not observed for the MC20CA0.5 formulations. The CA permeation profile from MC20CA0.5 was very similar at both temperatures, achieving an amount of drug permeated after 24 h equal to 41.19 and 34.02 μg cm^−2^, respectively, at 32 and 40 °C. The behaviour of MC20CA0.5 was due to the structural changes observed in the viscosity and the DSL studies, which are reported below. In fact, this formulation showed an important increase in the viscosity and size with the temperature increase. This trend was also related to the amount of drug retained in the skin layers after the permeation studies. In fact, the amount of CA that accumulated in the skin delivered by MC20CA0.5 within 24 h was one-and-a-half and two times higher than that delivered by MC10CA0.5, respectively, at 32 °C and 40 °C, indicating the ability of the micelles made up of the 20% P123 to form a drug depot in the skin layers (Figure 4), while, for the MC10CA0.5, the amount accumulated in the skin was the same at the two temperatures. 

### 2.3. Influence of Temperature on Rheological and DLS Studies

To better understand the different behaviours of the micelles in the permeation study with the temperature increment, investigations focused on the effect of temperature on the micelles’ size and rheological properties were carried out. DLS experiments were performed to investigate the effect of the temperature on the hydrodynamic diameter of the micelles, and the results are reported in Table 2.

The micellar size was observed to increase when heated, which revealed that the temperature alters the thermodynamics of micellisation and the assembly of the pluronics, as reported in the literature [27]. At a low temperature, water molecules predominantly form hydrogen bonds with the PEO blocks, whilst the PPO micelle core is mostly dehydrated. With a temperature increase, the hydration of the polymer chains decreases, and the block units undergo conformational changes, which results in a lower polarity. Consequently, the micelles grow in size and the morphology changes [28]. Moreover, this effecting result can be dependent on the polymer concentration [29,30].

**Figure 5:**
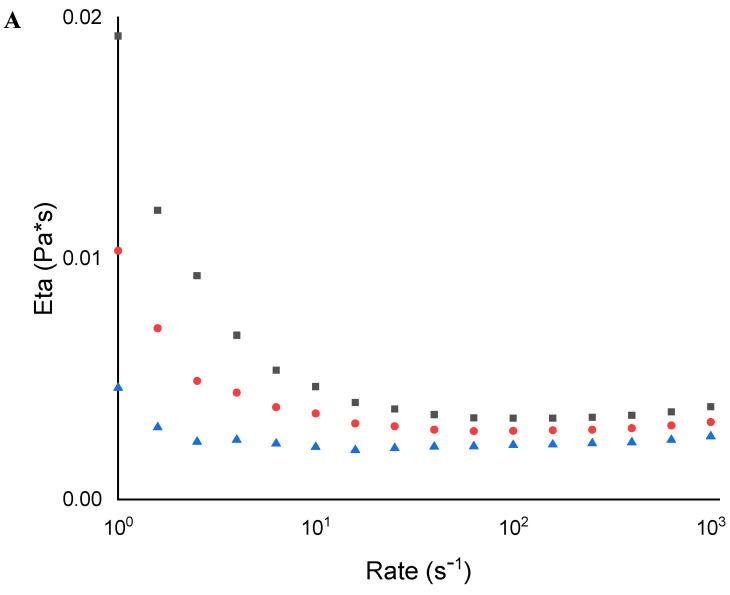

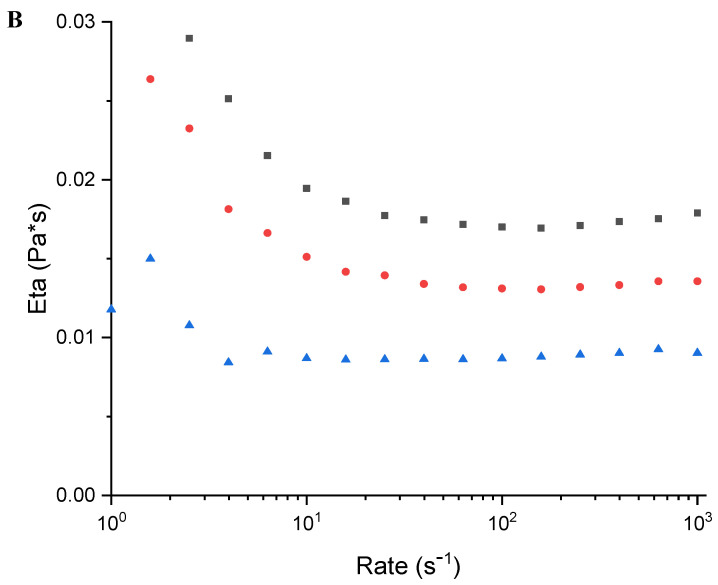
Rheological characterisation of (**A**) MC10 and (**B**) MC20 at 25 °C (■), 32 °C (●), and 40 °C (▲).

**Figure 6:**
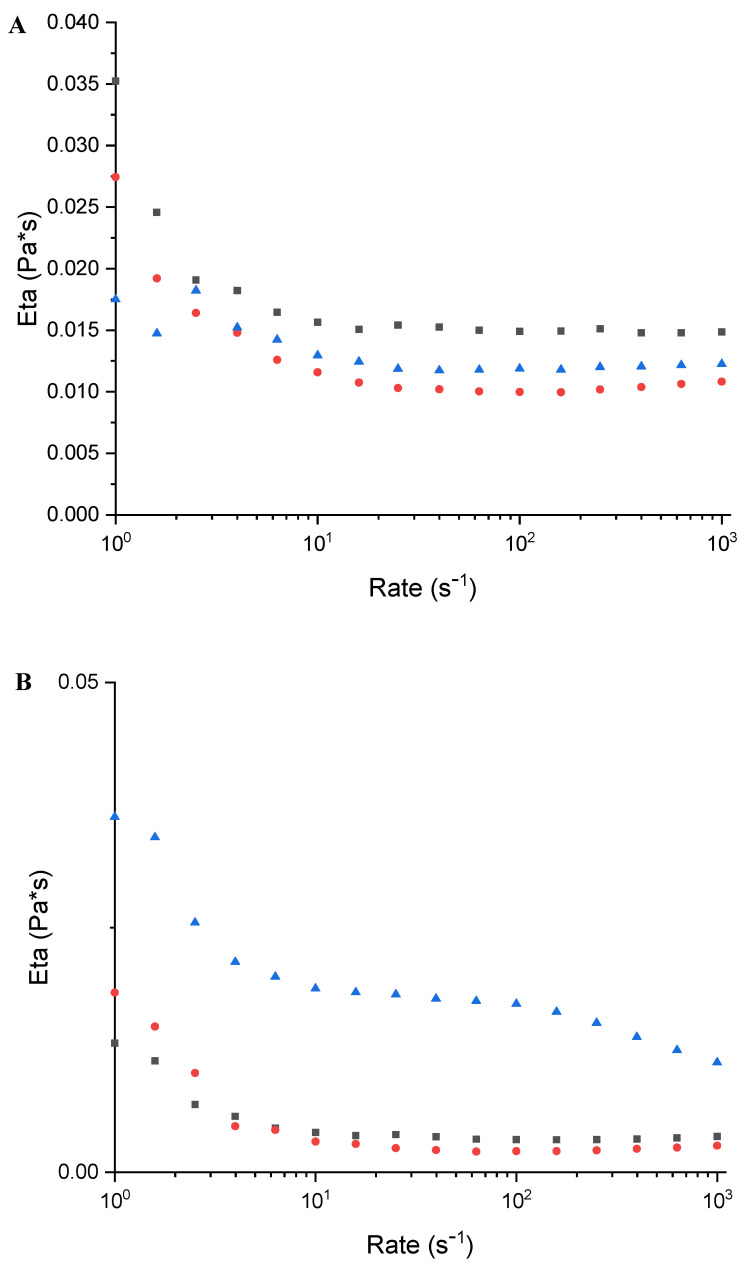
Rheological characterisation of (**A**) MC10CA0.5 and (**B**) MC20CA0.5 at 25 °C (■), 32 °C (●), and 40 °C (▲).

In addition, a rheological characterisation was carried out on the MC10 and MC20 micellar formulations, both empty and CA-loaded. Viscosity is a straightforward and effective method for tracking morphological changes in micellar solutions influenced by external stimuli. Steady-flow experiments (viscosity vs. shear rate) were performed in a shear rate range of 1–1000 s^−1^ (Figure 5 and Figure 6). To be sure that the steady flow moved into the samples, the flow equilibrium time was measured by transient experiments (the step-rate test), and it was observed that 10 s was a sufficient time to ensure the steady flow in the system for the overall investigated shear rate range. These experiments were conducted at three different temperatures: 25 °C, 32 °C, and 40 °C. The resulting studies showed that, for the empty micelle solutions (Figure 5), in both cases (10 wt% and 20 wt%), the viscosity decreased with the increasing temperature. It is worthy to note the initial shear thinning and low viscosity values. This means the micelles are small and lightly oblate [31]. The effect of the decreasing temperature was expected, owing to kinetic reasons. This thinning behaviour was slightly more pronounced with the increase in the P123 content, which also resulted in a higher viscosity. The micelles are, therefore, more oblate and bigger at a higher percentage of P123 [32].

When the CA is loaded in the micelles (Figure 6), the viscosity decreases at 32 °C; afterward, it increases at 40 °C. While this behaviour can be observed in both micelle solutions, at 20 wt%, this effect is dominant; in fact, the flow curves of the 20 wt% + CA solution are higher than those measured at 25 °C.

This means that, at 40 °C, the CA induces “gel formation”. In the presence of CA, the micelles grow and the shape becomes more asymmetrical.

From this behaviour, it can be assumed that the micelles have an oblate and not completely spherical shape. In addition, a characterisation of the size of MC20CA0.5 was conducted by means of a dynamic light-scattering analysis at different temperatures. Table 2 shows the values obtained, showing that, as the temperature increases, the average micelle diameter also increases.

### 2.4. Stability Studies

**Figure 7:**
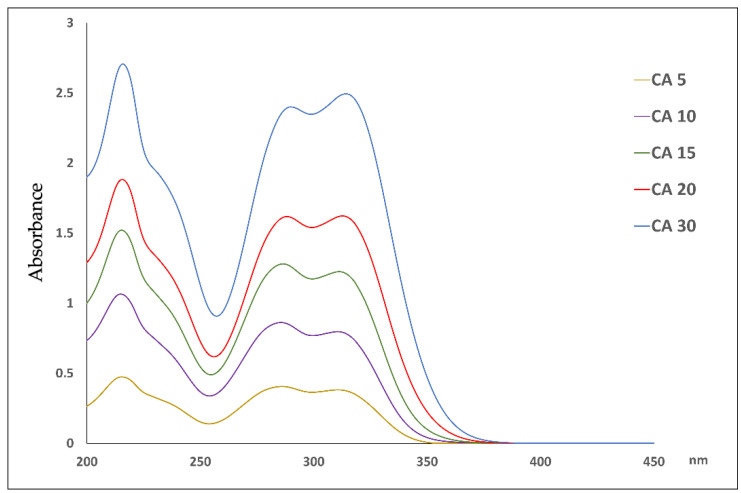
Absorbance spectra of CA aqueous solutions at different concentration values in the range of 5.0–30.0 μg mL^−1^.

The stability profile of the CA was assessed by exposing the aqueous solution of the compound and micelles to temperature and light. Thus, five solutions in the concentration range of 5.0–30.0 μg mL^−1^, corresponding to 28–167 μM, were prepared and subdued to the spectrophotometric measurement. The absorbance spectra of these solutions are depicted in Figure 7. Common to all phenolic substances, caffeic acid shows an intense absorption band at 280 nm, even at low concentration values. The use of a latest generation spectrophotometer allowed for high-performance results to be obtained for higher concentration solutions, which recorded absorbance values > one; in fact, the instrument can scan high absorbance values with a high accuracy (a high sensitivity up to six absorbance units).

In 2011, Abebe Belay and coll. [33] described the self-association of CA in water depending on the concentration of the solution due to the presence of hydrogen bonds on the catechol or carboxyl groups. These authors demonstrated the presence of a bathochromic shift of 2–7 nm, an intensity variation in the peaks, and three isosbestic points in the relationship with the increase in the concentration of the aqueous solution. When the concentration of CA is greater than 53.1 μM, the peak intensity at ~319 nm is greater than that of the two-peak band, but for concentrations less than 53.1 μM, the peak intensity at ~215 nm is greater than that of the doublet. In our measurements, the increase in the concentration, starting from 5.0 μg mL^−1^ to 30.0 μg mL^−1^, led to the shift of the peak at 215 to 216 nm, the one at 286 to 290 nm, and the one at 311 to 314 nm. Moreover, the ratio between the absorption maximum at 311–314 (λ_2_) and that at 286–290 (λ_1_) changed as the concentration of the solution increased. 

As reported in Table 3, the ratio A λ_2_/λ_1_ was calculated by selecting, in each experiment, the wavelength in which the absorbance corresponded to the maximum peak. Therefore, A λ_1_ and A λ_2_ were selected in the range of 286–290 and 311–314, respectively. The mathematical relationship between the concentration and the ratio A λ_2_/λ_1_ was calculated according to the following equation:

[A λ_2_/λ_1_] = 0.046 × [CA µg mL^−1^] + 0.8988    R^2^ = 0.8879

When the two maximum absorbance peaks had the same value, the ratio A λ_2_/λ_1_ was calculated to be equal to one and the concentration of CA was 22.0 µg mL^−1^. Above this concentration value, the presence of the self-association of CA is conceivable.

Stability tests were performed according to the ICH rules [25] to verify the effects of the temperature and light on the prepared solutions. The applied experimental conditions are described in Section 3.6 for both the experiments. The prepared five aqueous solutions of CA at the concentration values in the range of 5.0–30.0 μg mL^−1^ were exposed to light. Moreover, three aqueous solutions at the concentration values of 5.0, 20.0, and 30.0 μg mL^−1^ were exposed to thermal tests in thermostatic-controlled ovens at 60 and 80 °C, respectively. The UV spectra were recorded just after the preparation and at different interval times of stressed exposure for a total time of 120 min. The data matrix obtained from the collected spectra for each experiment was analysed by Multivariate Curve Resolution–Alternating Least Squares (MCR-ALS) to define the number of involved components (the number of degradation products), their absorbance spectra, and the kinetic rate of the degradation process (k values). The MCR method aims to describe the chemical contribution of each species formed in an experiment by decomposing the experimental data matrix (D) into a reduced set of contributions of chemical species (in our study, CA and its degradation products) by using a bilinear model. This model uses principal component analysis (PCA) algorithms to identify the number of components involved in the D matrix while excluding interferences that could be caused by experimental or instrumental noise. In the next step, the ALS algorithm uses a set of constraints to optimise the MCR model according to a chemical meaning. The quality and reliability of the model can be expressed in terms of the explained variance (%R^2^) and lack of fit (lof%). Herein, wavelengths below 200 nm were discarded as a preliminary selection due to their high variability or instrumental noise; thus, MCR processing was applied to spectral data between 200 and 450 nm in all experiments.

**Figure 8:**
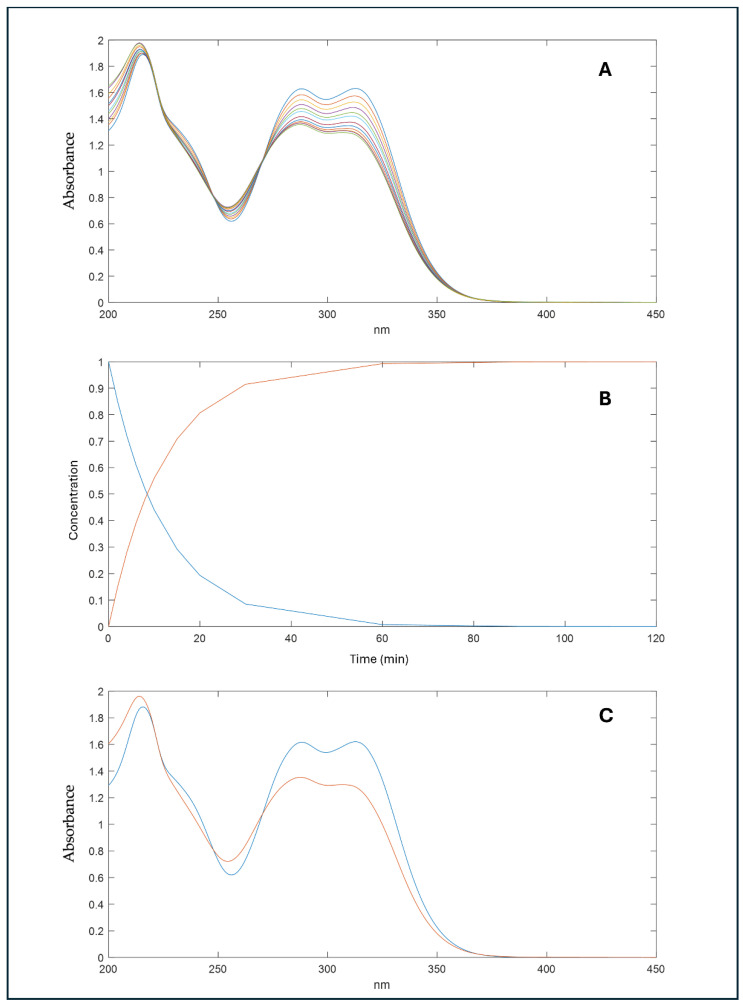
Photodegradation experiments of CA at a concentration of 20.0 μg mL^−1^ and spectral data processing by the MCR procedure with the formation of a single photoproduct. (**A**) Spectral sequences, (**B**) concentration profiles, (**C**) relative absorbance spectra of the pure compounds (blue line), and the photoproduct (red lines) obtained from the MCR elaboration. (**A**) Spectral sequence of CA recorded during the photodegradation experiment at different interval times, (**B**) concentration profile of the CA (blue line) and the relative photodegradation by-product (red line) obtained from the MCR analysis, (**C**) and UV spectra of the pure compound (blue line) and the relative photoproduct (red line).

When the CA was exposed to light, data processing by the MCR procedure showed the formation of a single photoproduct (CA-Ph) for all of the tested concentration values. As an example, Figure 8 shows the spectral sequence (part A) recorded during the photodegradation experiment on the CA solution at a concentration value of 20.0 μg mL^−1^. The MCR analysis allowed us to obtain the concentration profiles (part B) and the UV spectra of the pure compound and the relative photoproduct (part C).

A first-order kinetic equation was calculated in all of the photodegradation experiments, as follows:

ln [%CA] = −k × t + 4.67

where %CA is the percentage of the residual absorbance, k is the photodegradation rate, t is the time (s), and 4.67 is the logarithm of the initial absorbance (100%). In all of the experiments, the parameter lof%, which indicates the quality of the MCR results, was less than 4.1%, and the R^2^ was higher than 99.8%. Table 4 summarises the kinetic parameters calculated for the CA in different exposure conditions. The data were collected from three replicate analyses for each sample, and very low variance was measured in all of the cases. 

**Figure 9:**
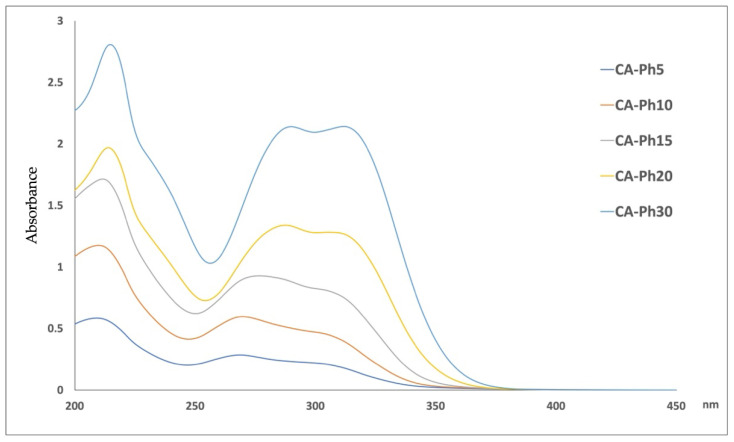
Absorbance spectra of the photodegradation products of the CA aqueous solutions at different concentration values in the range of 5.0–30.0 μg mL^−1^ obtained from the MCR data elaboration.

The spectra of the photodegradation products obtained by MCR after the photodegradation of the solutions prepared at different concentration values confirmed the presence of three absorption maximum peaks. The overlapping of these spectra, reported in Figure 9, showed that, in this case also, the shift of the absorption maximum was different based on the concentration of the solution. 

These shifts were measured at 209 to 215 nm and at 269 to 290. In addition, the peak at 312 nm shifted toward shorter wavelengths and flattened noticeably at low concentrations. This behaviour is also confirmed by the ratio between the two more important absorption maximums (269–290 and 312 nm) calculated for photodegradation products and reported in Table 3. The value of A λ_2_/λ_1_ was calculated to be equal to one for the sample in which the starting concentration of CA was 30.0 µg mL^−1^. The CA-Ph20 sample showed a A λ_2_/λ_1_ value below one according to the presence of the monomeric form of CA. The spectrum of the photodegradation product obtained from the MCR elaboration during the light exposure of the CA 30.0 μg mL^−1^ showed the same trend measured for the pure compound at 20.0 μg mL^−1^, as seen in the spectra reported in Figure S2.

The disappearance of the peak at 312 nm at a low concentration is also reported by Annaïg Le Person et al. [34], who investigated the kinetics and mechanism of trans-caffeic acid photodegradation, demonstrating intramolecular cyclisation with aesculetin formation after a longer irradiation time of CA from the 1000 W Xe light source. However, this route is not the only one, since the formation of vinylcatechol and protocatechuic acid seems to be important when the CA aqueous solution is irradiated with the Hg–Xe lamp. In our study, this second photodegradation profile could be supposed. The presence of a peak at 290 nm, the increase in the absorbance at 260 nm, and the disappearance of the peak at 312 nm, noticeable in Figure 8A, would seem compatible with the formation of protocatechuic acid, resulting in two maximum absorbance peaks at 260 and 290 nm [35].

**Figure 10:**
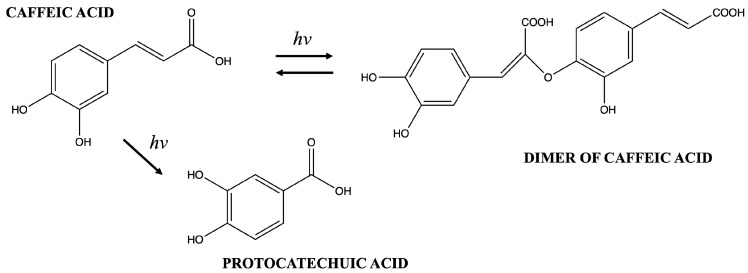
Proposed mechanism of the formation of degradation products after light exposure (*hv*).

These hypotheses about the mechanism of the formation of degradation products are described in Figure 10.

When the CA solutions were exposed to rising temperatures, no formation or degradation products were observed and the concentration of the pure compound was maintained up to 97% during all experiments.

**Figure 11:**
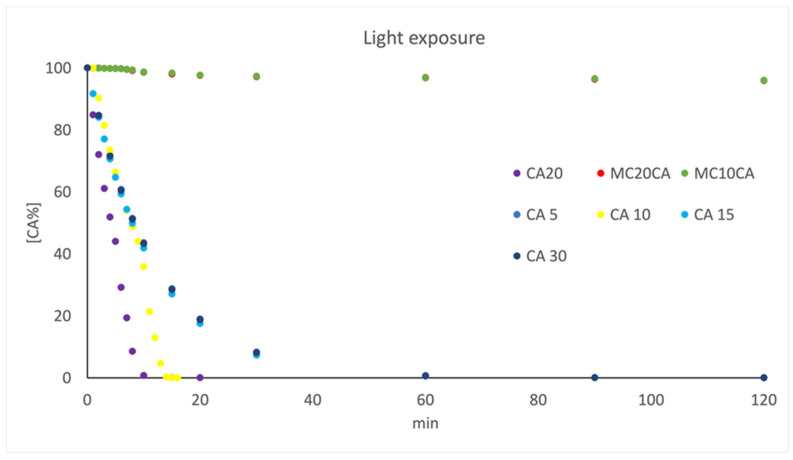
Kinetic profile of the photodegradation of the CA aqueous solution at different concentration values in the range of 5.0–30.0 μg mL^−1^, and CA micelles prepared with 0.5% of CA and 10 and 20% of surfactant.

The stability profile of the CA micelles were finally investigated. This prepared formulation was exposed to light and temperature in stress conditions. Stability during the increase in temperature was confirmed. Very promising results were obtained when the CA micelles were exposed to light, showing a residual concentration of the compound of 95% after 180 min of light exposure. Thus, the stability to light of CA was significantly improved compared to the aqueous solution in both formulations prepared with 10 and 20 surfactant concentrations percentages, respectively (Figure 11).

### 2.5. Antioxidant Properties

**Figure 12:**
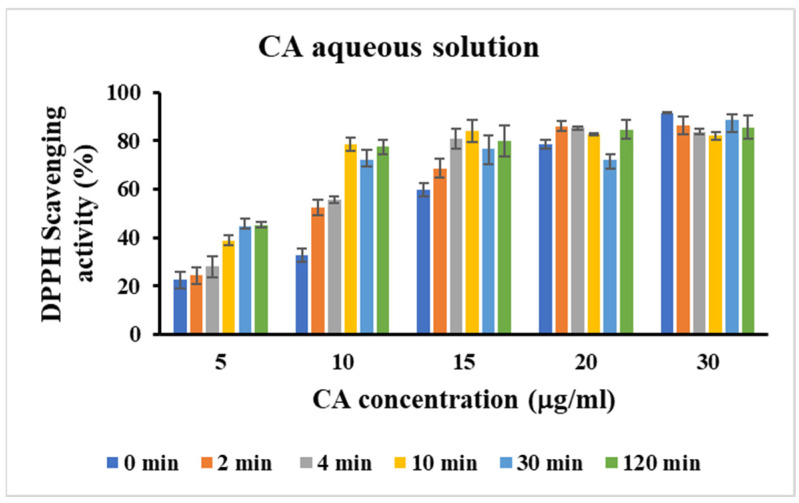
DPPH scavenging activity of CA aqueous solutions after the photodegradation experiment. Data are represented as mean ± SD; *n* = 3.

**Figure 13:**
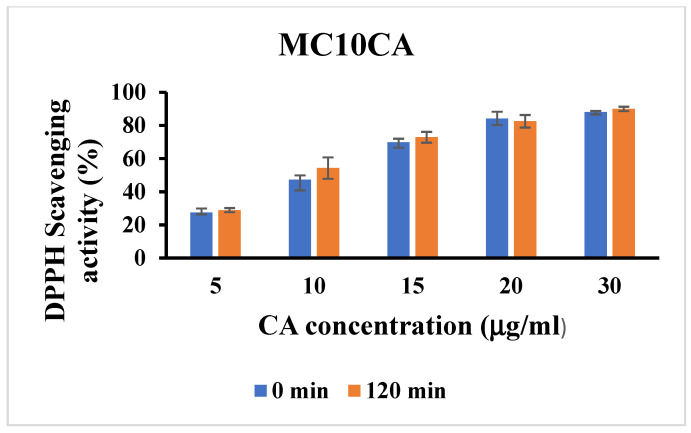
DPPH scavenging activity of MC10CA0.5 formulations after the photodegradation experiment. Data are represented as mean ± SD; *n* = 3.

To investigate whether CA retains its antioxidant properties when loaded in micelle systems, the DPPH scavenging activity of the formulations were evaluated and compared to free CA. Both free CA and CA loaded in micelle formulations displayed a significant concentration-dependent free radical scavenging activity. Moreover, no significant differences were observed for the antioxidant activity of the free CA and the one loaded in the micelles, which showed an amount required for the 50% inhibition of DPPH activity equal to 7.05 and 6.19 μg/mL, respectively. Therefore, our results showed that the loading of CA in micellar systems preserved its antioxidant activity. Moreover, the antioxidant activity of CA both as free and as loaded in micellar formulations after the stressed degradation experiment was also evaluated and compared with that before the degradation tests. As shown in Figures S3 and S4, the thermal stress tests carried out at 60 and 80 °C did not affect the antioxidant activity of free CA according to the degradation studies, which demonstrated that the free CA was stable in these conditions. On the contrary, the antioxidant activity of the free CA after the photodegradation experiments changed at the various time points (Figure 12). Above the starting concentration of 20.0 μg mL^−1^ CA, the solution showed that the maximum of the antioxidant activity was probably due to the presence of the dimeric form of the compounds. In fact, it is known that the dimer exhibits improved solubility properties and enhanced antioxidant properties compared to the parent compound [36]. Below this concentration value, the antioxidant activity of the degradation products resulted to be higher than that of the native molecule. This is probably related to the drug instability to light, which transforms it into a new photoproduct with a different antioxidant activity. It seemed that, even after subjecting a solution of CA to degradation, the solution still retained its DPPH radical scavenging activity. This suggests that the antioxidant properties of CA remained intact despite the degradation process. This behaviour could confirm the presence of protocatechuic acid as a photodegradation product, which exhibited an IC_50_ value of 1.88 μg mL^−1^ [37], which was much lower than the IC_50_ value of the CA, equal to 49.38 μg mL^−1^ [38]. Moreover, the increase in the antioxidant activity for a degradation product was already observed in a study of flavonoid compounds [39]. This finding could have implications for the potential use of CA or its derivatives in antioxidant-related applications, even under conditions that might typically degrade its structure. Nevertheless, the antioxidant activity of CA loaded in micelles was similar at each time point investigated during the photodegradation test, indicating the increased light stability of the drug when loaded in micellar formulations (Figure 13 and Figure S5).

## 3. Materials and Methods

### 3.1. Chemicals

Caffeic acid (CA) and 2,2-Diphenyl-1-picrylhydrazyl (DPPH) were purchased from Sigma Aldrich, Milan, Italy. The water was of pure grade. Pluronic P123, having a molar mass of 5800 gmol^−1^ with an average composition of (PEO)_20_(PPO)_70_(PEO)_20_, was purchased from Merck. The organic solvents used were supplied by VWR International SRL, Milan, Italy, and Sigma Aldrich.

### 3.2. Instruments and Software

Spectrophotometric measurements were conducted with the latest generation Perkin-Elmer Lambda 850+ UV/Vis Spectrophotometer (PerkinElmer, Boston, MA, USA). The scan rate was 1 nm s^−1^, the response time was 1 s, and the spectral band was 1 nm. UV spectra were registered in the range of 200–450 by means of the software UV Winlab^®^ 7.3.0.340 (PerkinElmer, Boston, MA, USA). According to the ICH rules, the photodegradation experiments were performed by using the Suntest XLS+ (Heraeus, Milan, Italy), equipped with a Xenon lamp (Atlas Material Testing Technology, Mt Prospect, IL, USA) and an ID65 light source (320 and 800 nm). Thermal stability tests were performed in a thermostatic-controlled oven (2100 High Performance Oven, produced by Fratelli Galli, Via dell’Artigianato, 12, 20072 Pieve Emanuele, Milano, Italy). The Multivariate Curve Resolution (MCR) algorithm was applied to the spectral data by using the Matlab^®^ computer environment software (Mathwork Inc., version 7, Natick, MA, USA).

### 3.3. Standard Solutions

Standard solutions of CA were prepared in water in the concentration range of 5.0–30.0 μg mL^−1^.

### 3.4. Preparation and Characterisation of Micelles

Empty and CA-loaded micelles were prepared by the direct dissolution method using an amount of P123 equal to 5%, 10%, and 20% (*w*/*w*) according to the phase diagram of P123 in aqueous solution [30]. An accurately weighted amount of P123 and CA, ranging from 0.1% to 5% (*w*/*w*), was solubilised in water and stirred at room temperature overnight to reach the thermodynamic equilibrium. The average particle hydrodynamic diameter, the polydispersity index (PI), and the surface charge of the resulting micelles were determined by light-scattering techniques using the Zetasizer Nano ZS 90 (Malvern Instrument, Worcestershire, UK). CA-loaded micelles were filtered through a 0.22 μm pore size syringe filter to remove the CA excess. The structure of the micelles was destroyed by dilution with ethanol, and the concentration of the CA solubilised in the final solution was evaluated using UV–Vis spectroscopy at 285 nm (absorbance maximum of CA) with the standard curve method. The encapsulation efficiency (EE%) was calculated using the following Equation:

$$\mathrm{EE}\%=\frac{\mathrm{CA}\;\mathrm{concentration}\;\mathrm{in}\;\mathrm{micelle}\;}{\mathrm{Initial}\;\mathrm{CA}\;\mathrm{concentration}}\times 100$$

All samples were analysed in triplicate

### 3.5. In Vitro Haemolysis Assay

Red blood cells were separated from heparinised blood by centrifugation at 3000 rpm for 10 min. Then, the plasma was removed, and the red blood cells were redispersed in PBS and washed three times. For the haemolysis assay, an erythrocyte suspension was diluted in PBS to 8 × 10^9^ cells/mL. An aliquot of 25 mL of the erythrocyte suspension was mixed with various volumes of micelles in microtubes, and the final volume was completed to 1 mL with PBS at a pH of 7.4. The mixtures were incubated under shaking at room temperature for 10 min. At the end of the incubation, the samples were centrifuged at 10,000 rpm for 5 min. The absorbance of the haemoglobin release in the supernatants was measured at 540 nm using a UV–Vis spectrophotometer, and the percentages of the haemolysis were determined by comparison with the positive control samples completely haemolysed with Triton-X 100.

### 3.6. Experimental Conditions in the Stability Studies

Five aqueous solutions of CA were prepared at the following concentration values: 5.0, 10.0, 15.0, 20.0, and 30.0 μg mL^−1^, and directly light-irradiated in the Suntest cabinet. The irradiance power was fixed to 350 W m^−2^, corresponding to a light dose of 21 kJ min^−1^ m^−2^; the temperature was about 25 °C. Three aqueous solutions were exposed to thermal tests in a thermostatic bath at 60 and 80 °C, respectively. For each experiment, the UV spectrum was recorded just after the preparation and at the following interval times of exposure: 1, 2, 3, 4, 5, 6, 7, 8, 10, 15, 20, 30, 60, 90, and 120 min. The data matrix obtained from the collected spectra was analysed by MCR-ALS in order to define the number of the involved components (the number of degradation products), their absorbance spectra, and the kinetic rate of the degradation process (k values). 

### 3.7. Radical Scavenging Activity

Stock dispersions of CA free and loaded in micelles were diluted in order to obtain different concentrations. An amount of 1.5 mL of the sample was incubated with 1.5 mL of the ethanol DPPH solution, totalling 0.25 mM, at room temperature in the dark. After 30 min, absorbance measurements were taken at 517 nm with a UV–Vis spectrophotometer. The DPPH radical scavenging activity was calculated according to the following equation:

DPPH Scavenging activity (%) = (A_0_ − A_1_)/A_0_ × 100 (1)

where A_0_ is the absorbance of the control and A_1_ is the absorbance in the presence of the free CA and CA loaded in micelle formulations. Moreover, the antioxidant activity of the degradation products of caffeic acid was estimated after the photodegradation process. Each experiment was carried out in triplicate and the results were expressed as means ± SD.

### 3.8. In Vitro Diffusion and Skin Permeation Studies

In vitro CA diffusion and skin permeation from the P123 micelles was evaluated by Franz cells at two different temperatures, 32 and 40 °C. Particularly, the diffusion barrier was made with Visking tubing (Spectra/Por^®^, cut-off 12–14 kDa), while, for the permeation studies, rabbit ear skin was used. The artificial membrane and skin were clamped between the donor and the receptor compartment of the Franz diffusion cell. The effective penetration area was 0.416 cm^2^ and the receptor compartment capacity was 5.5 mL. The receptor compartment was filled with medium and, at various time points, the medium in the receptor compartment was collected and replenished immediately with an equal amount to maintain the sink conditions. Then, the CA concentrations released were quantified using UV–Vis spectroscopy. The cumulative amount of CA permeated per unit area was calculated, and the permeation profile was plotted as a function of time. In order to determine the amount of CA retained on the skin, the skin was carefully separated from the diffusion cell at the end of the skin permeation experiments (24 h). Then, it was rinsed with deionised water to remove any residual formulation. Then, the retained CA on the skin was extracted by soaking it in 10 mL of ethanol for 1 h with constant stirring in the dark. The solution was then filtered by a membrane (0.22 μm), and the content of CA was analysed by UV–Vis spectroscopy.

## 4. Conclusions

In summary, polymeric micelles of caffeic acid were prepared using pluronic P123 at two different concentrations to enhance its solubility, bioavailability, and stability. Loading CA within the micelles significantly increased its water solubility, resulting in formulations with high colloid stability suitable for various routes of application. The polymer concentration and temperature affected the drug diffusion and permeation. More importantly, the micelles developed effectively protected the CA from degradation when exposed to light, offering significant benefits for various biomedical applications. Overall, the use of pluronic P123 micelles as carriers for caffeic acid encapsulation holds promise as an approach to address the issues of poor solubility and stability, paving the way for the development of safer, cost-effective delivery systems with enhanced therapeutic potential.

## Figures and Tables

**Table 1:** Physicochemical characterisation of empty (MC) and CA-loaded micelles (MCCA). P123 solutions at 5, 10 and 20% were used to prepare the MC5, MC10, and MC20, both empty and CA-loaded, respectively.

Sample	P123% (*w*/*w*)	CA% (*w*/*w*)	Size ± S.D. (nm)	PI	Z Potential (mV)	EE%
MC5	5	-	16.09 ± 0.23	0.113	−6.12 ± 0.23	-
MC5CA0.1	5	0.1	17.37 ± 0.16	0.218	−5.72 ± 1.07	77.68
MC5CA0.2	5	0.2	16.47 ± 0.02	0.138	−4.10 ± 2.11	82.59
MC10	10	-	13.37 ± 0.45	0.193	−4.81 ± 1.66	-
MC10CA0.5	10	0.5	16.04 ± 0.92	0.180	−1.08 ± 0.18	78.73
MC20	20	-	15.16 ± 0.56	0.214	−2.12 ± 0.35	-
MC20CA0.5	20	0.5	12.16 ± 1.12	0.217	−1.62 ± 0.42	80.55

**Table 2:** DLS analysis of micelle formulations with increasing temperatures from 25 °C to 44 °C.

	MC10CA0.5		MC20CA0.5	
	Size (nm)	PI	Size (nm)	PI
25 °C	16.04	0.180	12.16	0.217
29 °C	16.80	0.195	12.41	0.210
32 °C	22.78	0.340	13.82	0.271
35 °C	30.36	0.393	19.13	0.458
38 °C	35.40	0.369	19.13	0.558
41 °C	37.75	0.367	26.54	0.529
44 °C	41.58	0.373	27.52	0.470

**Table 3:** Absorbance values of the maximum peaks of CA solutions.

[CA µg mL^−1^]	A λ_1_ 286–290 nm	A λ_2_ 311–314 nm	A λ_2_/λ_1_
CA 5	0.407	0.382	0.940
CA 10	0.862	0.798	0.925
CA 15	1.280	1.225	0.957
CA 20	1.618	1.622	1.003
CA 30	2.400	2.493	1.039
	269–290 nm	312 nm	
CA-Ph20	1.338	1.281	0.957
CA-Ph30	2.139	2.141	1.001

**Table 4:** Kinetic photodegradation parameter calculated for the CA in solutions at different concentration values (k: photodegradation rate; lof%: lack of fit %; R^2^: determination coefficient).

[CA µg mL^−1^]	k × 10^−3^	lof %	R^2^
5.0	1.503	4.071	99.834
10.0	1.710	1.042	99.989
15.0	1.451	0.879	99.992
20.0	1.368	0.657	99.996
30.0	1.507	0.355	99.999

## Data Availability

The data presented in this study are available upon request from the corresponding author.

## Supplementary Materials

The following supporting information can be downloaded at: https://www.mdpi.com/article/10.3390/molecules29143329/s1, Figure S1. Intensity size distribution of MC10CA0.5 (A) and MC20CA0.5 (B) determined by DLS; Figure S2. Absorbance spectra of CA 20 µg mL^−1^ (red line), CA 20 µg mL^−1^ (grey line) and the photodegradation product obtained for CA 30 µg mL^−1^ (blue line); Figure S3. DPPH scavenging activity of CA aqueous solutions after thermal degradation experiment carried out at 60 °C; Figure S4. DPPH scavenging activity of CA aqueous solutions after thermal degradation experiment carried out at 80 °C; Figure S5. DPPH scavenging activity of MC20 formulations after photodegradation experiment. Data is represented as Mean ± SD, n = 3.
