# Supplementary material for: Formulation of Polymeric Micelles to Increase the Solubility and Photostability of Caffeic Acid
Source: Molecules. 2024 Jul 15;29(14):3329. doi: 10.3390/molecules29143329 (PMC11279489; doi:10.3390/molecules29143329)
Supplement: Supplementary file 1 [file molecules-29-03329-s001.zip › molecules-3090281-supplementary.pdf]

Formulation of polymeric micelles to increase the solubility and photostability of caffeic acid

Elisabetta Mazzotta[1], Martina Chieffallo[1], Rita Muzzalupo[1*], Miriana Spingola[1], Paolino Caputo[2], Martina Romeo[1], Giuseppina Ioele[1].

[1]Department of Pharmacy, Health and Nutritional Sciences, University of Calabria, 87036 Rende, Italy;
[2]Department of Chemistry and Chemical Technologies, Cubo 14/D, University of Calabria, Via P. Bucci, 87036 Arcavacata di Rende, Italy;
[*]Corresponding author: rita.muzzalupo@unical.it

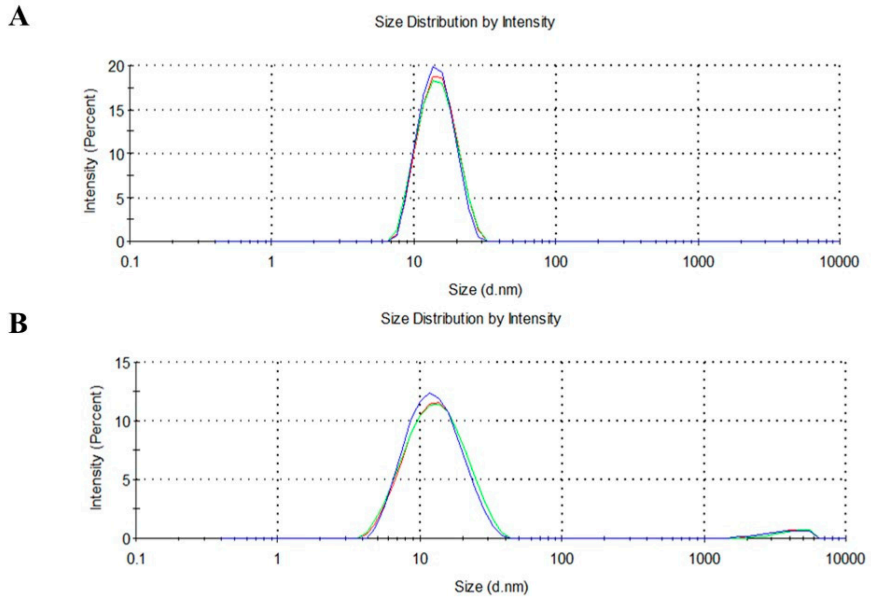

Figure S1. Intensity size distribution of MC10CA0.5 (A) and MC20CA0.5 (B) determined by DLS.

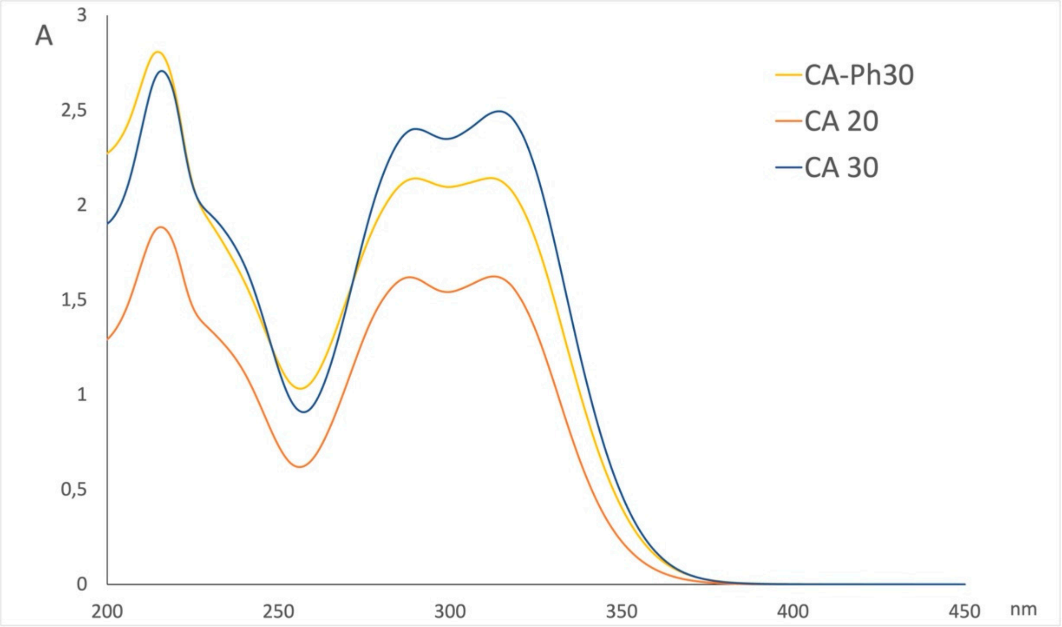

Figure S2. Absorbance spectra of CA 20 µg mL^{-1} (red line), CA 20 µg mL^{-1} (grey line) and the photodegradation product obtained for CA 30 µg mL^{-1} (blue line).

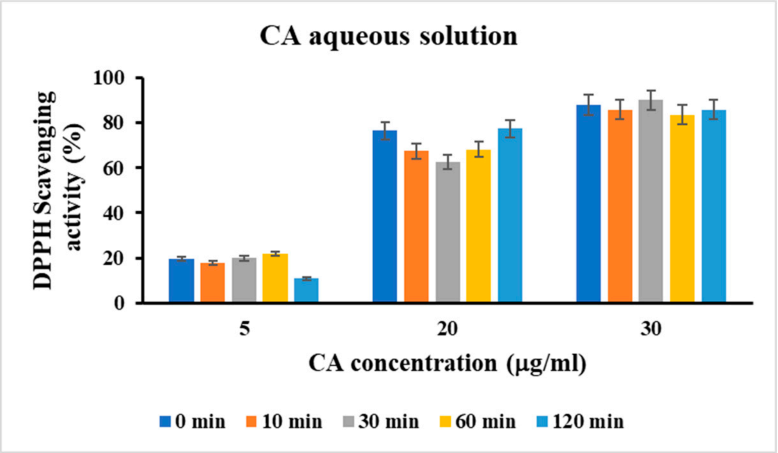

Figure S3. DPPH scavenging activity of CA aqueous solutions after thermal degradation experiment carried out at 60°C

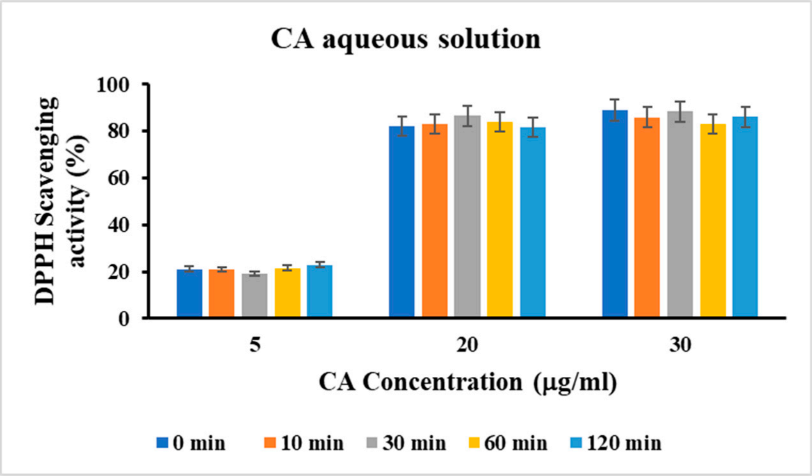

Figure S4. DPPH scavenging activity of CA aqueous solutions after thermal degradation experiment carried out at 80°C

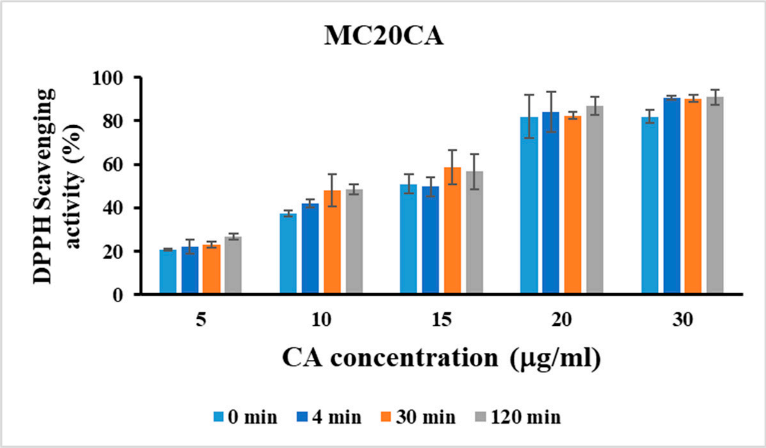

Figure S5. DPPH scavenging activity of MC20 formulations after photodegradation experiment. Data is represented as Mean ± SD, n=3.
